# MOF-Derived Spindle-Shaped Z-Scheme ZnO/ZnFe_2_O_4_ Heterojunction: A Magnetic Recovery Catalyst for Efficient Photothermal Degradation of Tetracycline Hydrochloride

**DOI:** 10.3390/ma16206639

**Published:** 2023-10-11

**Authors:** Shilong Suo, Wenmei Ma, Siyi Zhang, Ziwu Han, Yumin Wang, Yuanyuan Li, Yi Xiong, Yong Liu, Chunqing He, Pengfei Fang

**Affiliations:** 1 Key Laboratory of Nuclear Solid State Physics Hubei Province, School of Physics and Technology, Wuhan University, Wuhan 430072, China; 2022202020075@whu.edu.cn (S.S.);; 2 Department of Microelectronics, School of Mathematical & Physical Sciences, Wuhan Textile University, Wuhan 430073, China

**Keywords:** Z-scheme, tetracycline, photocatalysis, ZnFe_2_O_4_, ZnO

## Abstract

The development of photocatalysts with a wide spectral response and effective carrier separation capability is essential for the green degradation of tetracycline hydrochloride. In this study, a magnetic recyclable Z-scheme ZnO/ZnFe_2_O_4_ heterojunction (ZZF) was successfully constructed via the solid phase method, using MIL-88A(Fe)@Zn as the precursor. An appropriate band gap width and Z-scheme charge transfer mechanism provide ZZF with excellent visible light absorption performance, efficient charge separation, and a strong redox ability. Under visible light irradiation, the degradation efficiency of tetracycline hydrochloride for the optimal sample can reach 86.3% within 75 min in deionized water and 92.9% within 60 min in tap water, exhibiting superior stability and reusability after five cycles. Moreover, the catalyst in the water can be conveniently recovered by magnetic force. After visible light irradiation for 70 min, the temperature of the reaction system increased by 21.9 °C. Its degradation constant (35.53 × 10^−3^ min^−1^) increased to 5.1 times that at room temperature (6.95 × 10^−3^ min^−1^). Using thermal energy enhances the kinetic driving force of the reactants and facilitates carrier migration, meaning that more charge is available for the production of •O_2_^−^ and •OH. This study provides a potential candidate for the efficient degradation of tetracycline hydrochloride by combining thermal catalysis with a photocatalytic heterojunction.

## 1. Introduction

Antibiotics are widely used to promote plant growth and control disease outbreaks. Tetracycline hydrochloride (TCH), a broad-spectrum antibiotic, is used in disease treatment and the aquaculture industry [1]. Nevertheless, it cannot be fully metabolized in living organisms, which leads to an increase in the residual amount of TCH in the environment [2]. In recent years, TCH and its metabolites have been detected in surface water, groundwater, and drinking water [3,4,5]. At present, antibiotics represented by TCH are one of the most significant pollutants in water [6]. Therefore, it is necessary to establish a feasible solution to degrade TCH in water.

Photocatalysis technology is currently the most promising approach for the degradation of TCH because it is environmentally friendly and inexpensive [7,8]. The photocatalysis reaction involves the absorption of photons with energy greater than that of the semiconductor band gap. This process produces electron–hole pairs, which then migrate to the surface to form active substances [9], such as •O_2_^−^, •OH, etc. This may lead to two main bottlenecks. The first is that some semiconductor photocatalysts with wide band gaps (such as TiO_2_, ZnO, etc.) can only absorb ultraviolet light, which accounts only for 5% of the total solar spectrum [10]. The second is the easy recombination of photogenerated carriers [11,12,13]. A variety of strategies, including element doping and heterojunction construction, have been developed to solve these problems [14,15,16,17,18,19,20,21,22]. Among these strategies, the construction of a heterojunction is a research hotspot [23]. For example, a variety of heterojunction photocatalysts [16], including type I [24], type II [25], Z-scheme [26,27], and S-scheme [10] heterojunctions, have been extensively studied with regard to the visible-light degradation of TCH. On the other hand, collaborative photocatalysis technology (such as thermo-assisted photocatalysis) is also an effective means [13]. The introduction of thermal energy can facilitate the separation and migration of charge carriers [28,29,30], accelerate mass transfer, enhance kinetic driving force [13,31], promote the dissociation of reactant molecules, and adjust the redox potential of half reactions [32,33]. Thus far, thermo-assisted photocatalysis has been widely used in CO_2_ reduction [28,32], hydrogen evolution [34], pollutant degradation [35,36], organic synthesis [37,38], and many other processes.

In addition, the recovery technology of photocatalysts from liquid-phase reaction systems is also a major obstacle to their practical application, especially for those very fine and small particulate catalyst solids [39,40]. Recently, magnetic photocatalysts have attracted much attention because they have greatly simplified postreaction processing and catalyst recycling by simply applying a magnetic field [41,42]. ZnFe_2_O_4_ is a common magnetic semiconductor with a narrow band gap (≈1.9 eV), low cost, and excellent photochemical stability [43,44,45]. Its magnetic properties make it easy and economical to recover from the liquid phase [46]. Additionally, magnetism may also change the spin polarization of electrons and promote the separation of electron–hole pairs [43]. The Fe element in ZnFe_2_O_4_ has the potential to be used in the Fenton reaction [45,47]; therefore, it is attracting increasing attention from researchers [48,49,50,51]. For example, Fei et al. [52]. synthesized ZnFe_2_O_4_/In_2_O_3_ Z-scheme heterojunctions. Their excellent carrier separation efficiency gives them excellent photocatalytic activity and stability during TCH degradation. Yang et al. [53] used the intrinsic catalytic behavior of ZnFe_2_O_4_ (similar to peroxidase) to efficiently and consistently convert hydroxyl benzene into phenol.

In summary, a magnetic recyclable ZnO/ZnFe_2_O_4_ Z-scheme heterojunction with excellent photothermal conversion capability was prepared and designed. The morphology, structure, and chemical states of the elements of the prepared photocatalyst were studied using scanning electron microscopy (SEM), high-resolution transmission electron microscopy (HRTEM), X-ray diffraction (XRD) patterns, and X-ray photoelectron spectroscopy (XPS) tests. Subsequently, we systematically evaluated the photothermal catalytic activity and stability of the prepared ZZF heterojunction. The effects of the quality of ions in water and pH on catalytic performance were determined via comparative experiments. As expected, the ZZF photocatalyst had excellent photothermal catalytic performance and stability. The band structure was studied using Mott–Schottky (M-S) plots and UV–visible diffuse reflectance (DRS) spectra. The main active species were ascertained via the terephthalic acid photoluminescence (TA-PL) experiment, electron spin resonance (ESR) test, and free radical capture experiment. Combined with the band structure and the active substances produced during the reaction, the carrier transfer pathways were determined as the Z-scheme mechanism. This study aims to provide ideas for designing photothermal catalysts with excellent antibiotic degradation performance.

## 2. Materials and Methods

**Figure 1:**
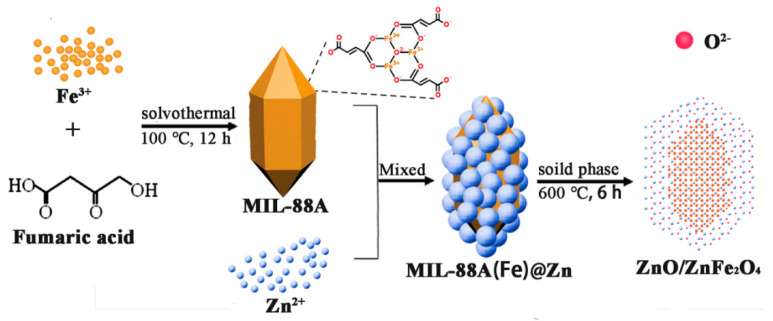
Schematic illustration for the preparation process of the ZnO/ZnFe_2_O_4_ heterojunction.

The materials and their characterization are fully described in the Supplementary Materials (Texts S1 and S2). The schematic illustration for the preparation process of the ZnO/ZnFe_2_O_4_ heterojunction is shown in Figure 1. Additionally, the methods for the photoelectrochemical test, photothermal conversion performance, and hydroxyl radical concentration test are listed in the Supplementary Materials as Texts S3–S5.

### 2.1. Preparation of MIL-88A(Fe)

MIL-88A(Fe) was synthesized using a modified method reported in [54]. Firstly, 10 mmol FeCl_3_·6H_2_O and 10 mmol C_4_H_4_O_4_ were dissolved in 50 mL DMF. Subsequently, the mixed solution was heated at 100 °C for 12 h in a 100 mL reactor, and then the precipitate was washed with DMF and ethanol several times. Eventually, the resulting product was soaked in deionized water (DI water) for 12 h to remove residual DMF and then dried at 120 °C for 12 h.

### 2.2. Preparation of ZnO/ZnFe_2_O_4_ Heterojunction

A certain amount of MIL-88A(Fe) and 20 mmol Zn(NO_3_)_2_·6H_2_O were added to 15 mL DI water, and then all water was evaporated under continuous stirring to prepare MIL-88A(Fe)@Zn. Subsequently, MIL-88A(Fe)@Zn was heated in a tube furnace at a heating rate of 5 °C/min to 600 °C and held for 6 h to obtain a ZnO/ZnFe_2_O_4_ heterojunction. When the dosages of the added MIL-88A(Fe) were 40 mmol, 20 mmol, and 10 mmol, the prepared ZZF heterojunctions were recorded as ZZF1, ZZF2, and ZZF3, respectively. For comparison, ZnFe_2_O_4_ (ZFO) was prepared by adding 43 mmol MIL-88A(Fe). ZnO was obtained by the calcination of Zn(NO_3_)_2_·6H_2_O also at a heating rate of 5 °C/min to 600 °C and held for 6 h.

### 2.3. Photocatalytic and Photothermal Catalysis Activity Measurements

Typically, 20 mg of the catalyst was dispersed into 50 mL of TCH aqueous solution (100 mg/L). The mixture was stirred in the dark for 30 min to reach the adsorption–desorption equilibrium prior to light irradiation; the suspension was irradiated under a Xenon lamp (300 W) coupled with an optical cut-off filter (λ ≥ 400 nm). Then, 1 mL of the reaction solution was collected at intervals of 15 min. Subsequently, the obtained solution was centrifuged at 12,000 rad/min for 1 min, and 0.8 mL supernatant was taken, diluted with 0.8 mL deionized water, and analyzed using a UV–Vis spectrophotometer. The temperature of the system was controlled using additional circulating water in the process of photothermal catalysis.

## 3. Results and Discussion

### 3.1. Morphology and Structure

**Figure 2:**
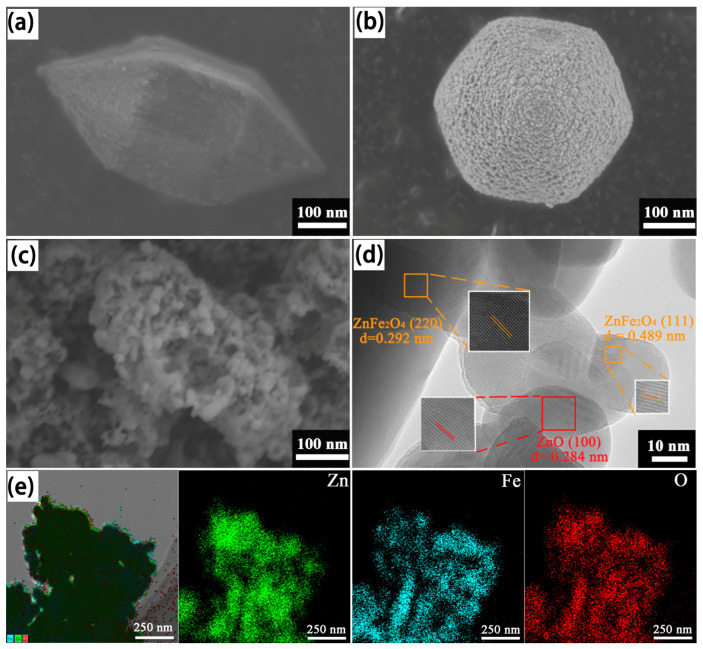
SEM images of (**a**) MIL-88A(Fe), (**b**) MIL-88A(Fe)@Zn, and (**c**) ZZF2; HRTEM images of (**d**) ZZF2; (**e**) EDX mapping results of Zn, Fe, and O of the ZZF2.

To investigate the morphology and structure of the ZnO/ZnFe_2_O_4_ heterojunction, ZZF2 and its precursors were analyzed using SEM and HRTEM. As shown in Figure 2a, MIL-88A (Fe) has a spindle-shaped structure with a length and width of about 500 nm and 300 nm. After MIL-88A(Fe) is mixed with zinc nitrate, zinc nitrate is relatively uniform and attaches to the surface of MIL-88A(Fe) to form MIL-88A(Fe)@Zn (Figure 2b) [43]. Figure 2c shows that the surface of the ZZF2 sample becomes rough after annealing, and the spindle structure is slightly damaged. Figure 2d shows that the ZZF2 sample has significant lattice fringes with crystal fringe spacing of 0.292 nm, 0.489 nm, and 0.284 nm, which perfectly corresponds to the (220), (111) crystal faces of ZnFe_2_O_4_ and the (100) crystal faces of ZnO, respectively. The EDX spectra (Figure S1a) and EDX mapping (Figure 2e and Figure S1b) of the ZZF2 composite verified the presence of Zn, Fe, and O elements. The thermogravimetric curve of MIL-88A (Fe)@Zn (Figure S1c) shows that the weight of the precursor steadily decreases with increasing temperature. This means that the adsorbed water, C, and N in the precursor are progressively released. When the temperature reaches 500 °C, the mass does not change, proving that the precursor has transformed into a stable inorganic substance. The EDX mapping shows that the distribution of Zn and Fe elements in the ZZF2 is not synchronous, indicating that ZnO/ZnFe_2_O_4_ heterostructures have been successfully constructed.

**Figure 3:**
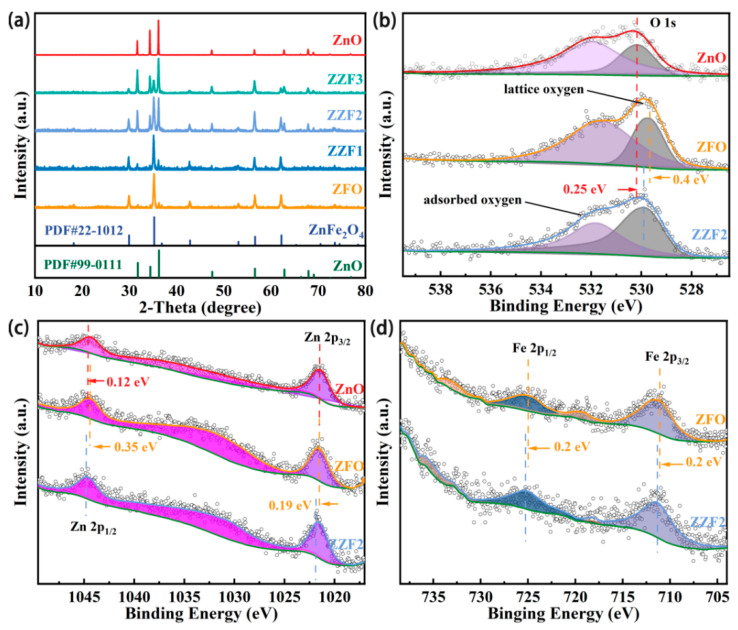
(**a**) XRD patterns of ZnO, ZZF samples, and ZnFe_2_O_4_. XPS spectra related to (**b**) O 1s, (**c**) Zn 2p, (**d**) Fe 2p of ZnFe_2_O_4_, ZZF2, and ZnO samples, respectively.

As shown in Figure 3, the crystal phase and crystallinity of prepared samples are determined using XRD patterns. The peaks of hexagonal wurtzite ZnO at 2θ values of 31.76°, 34.42°, 36.25°, 47.54°, 56.5°, 62.85°, 67.94°, and 69.08° refer to the *hkl* indices (100), (002), (101), (102), (110), (103), (112), and (201), respectively (PDF#99-0111). Similarly, the peaks of cubic spinel crystal ZnFe_2_O_4_ are observed as 2θ at 29.82°, 35.21°, 42.69°, 53.32°, 56.52°, and 62.12°, which are the indices of the (220), (311), (400), (422), (511), and (440) planes, respectively (PDF#22-1012). The diffraction peaks of ZnO and ZFO simultaneously appear in samples ZZF1, ZZF2, and ZZF3, and the positions of the peaks do not shift. This shows that the crystal lattice of the semiconductor is not destroyed when the heterojunction is formed. This is advantageous for photogenerated carrier migration. With the increase in Zn element content, the peak strength representing ZnO gradually increases and the peak intensity representing ZFO gradually decreases in the XRD spectra. All peaks are sharp, indicating that the synthesized samples have excellent crystallinity.

The surface element composition and chemical states of the ZnO, ZZF2, and ZFO were further characterized by XPS. All binding energy (BE) values were calibrated via a C 1s peak at 284.8 eV. Figure S2 shows that Zn, Fe, and O elements all exist in the ZZF2 sample. Additionally, two convolution peaks can be fitted in the O 1s spectra in Figure 3b. These two peaks represent surface-adsorbed oxygen (at about 531.5 eV) and lattice oxygen (at about 529.7 eV), respectively [43,44]. In the high-resolution spectra of Zn 2p, the BE of Zn 2p_3/2_ changes from 1021.63 eV (ZnO) and 1021.46 eV (ZFO) to 1021.65 eV (ZZF2), respectively. Similarly, the BE of Zn 2p_1/2_ shifts from 1044.59 eV (ZnO) and 1044.36 eV (ZFO) to 1044.71 eV (ZZF2), respectively. The BE of Fe 2p_3/2_ and Fe 2p_1/2_ in ZFO are 711.11 eV and 724.91 eV, respectively, corresponding to the characteristic peak of Fe^3+^. After forming the ZZF2 sample, the BE shifts to 711.51 eV (Fe 2p_3/2_) and 725.11 eV (Fe 2p_1/2_), respectively. The BE of Zn and Fe elements in the ZZF2 sample are slightly higher than those in ZnO and ZFO. The change in chemical state further verifies the successful synthesis of the heterojunction [18,43].

Figure S3 shows the N_2_ adsorption–desorption isotherms and pore-size distribution curves of the ZZF2 sample. According to IUPAC classification, N_2_ absorption/desorption isotherms in the ZZF2 sample belong to type-IV with an H3 hysteresis loop, and the distribution of the majority of pores is 2–10 nm, manifesting the presence of mesopore structures [55].

### 3.2. Photocatalytic Activity and Reusability

**Figure 4:**
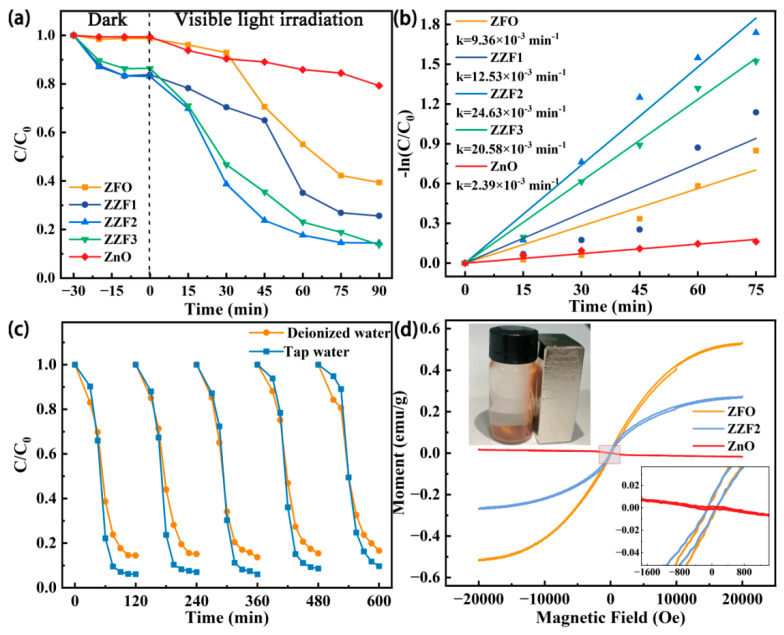
(**a**) Photocatalytic performance of TCH degradation, and (**b**) corresponding pseudo-first-order kinetics curves of prepared samples. (**c**) Cycling experiments of photocatalytic TCH degradation of ZZF2 in DI water and tap water. (**d**) Magnetic hysteresis loops of ZnO, ZnFe_2_O_4_, and ZZF2.

Through the experimental study of the photocatalytic degradation of TCH under visible light irradiation, the activity of the prepared photocatalysts was evaluated (Figure 4a). The degradation efficiency values of ZZF1, ZZF2, and ZZF3 for TCH are higher than those of pure ZnO and ZFO, indicating that the construction of a heterojunction promotes the degradation activity of TCH. The ZZF2 samples showed the best photocatalytic activity, and the degradation rate reached 86.3% after 75 min of visible light irradiation. Figure 4b shows the reaction kinetics (k_TCH_) of the prepared photocatalysts, and the experimental data conform to pseudo-first-order kinetics [43,51]:

ln(C_0_/C) = kt (1)

The k_TCH_ values of ZFO, ZZF1, ZZF2, ZZF3, and ZnO are 9.36 × 10^−3^ min^−1^, 12.56 × 10^−3^ min^−1^, 24.63 × 10^−3^ min^−1^, 20.58 × 10^−3^ min^−1^, and 2.39 × 10^−3^ min^−1^, respectively. The k_TCH_ value of ZZF2 is 2.63 times that of ZFO and 10.3 times that of ZnO. Compared with other photocatalysts, the degradation effect is still at its highest level (Table S1).

The cyclic stability of ZZF2 in DI water was studied (Figure 4c). After five cycles, the photocatalytic activity of ZZF2 on TCH in DI water decreased to 80% and maintained 92.6% of its maximal activity. Further research was conducted on the ZZF2 sample’s catalytic activity in tap water. The results show that the degradation efficiency reached 92.9% in 60 min, which is greater than the value recorded for DI water. The efficiency remained at 83.74% after five cycles. The properties decay slightly faster than in DI water, leaving only 90.1% of the original solution. The XRD results demonstrate that the crystal structure of the ZZF2 sample has no significant change after the reaction (Figure S4). In conclusion, the ZZF2 sample has good photocatalytic activity and cycle stability.

We found that the degradation effect of ZZF2 on TCH in tap water was better than that in DI water. The difference between tap water and DI water is ion content and pH. The main ions in tap water are listed in Table S2. Compounds were added to the prepared 100mg/L TCH solution so that it contained the same concentration of ions as tap water. To determine the degradation effect of ions in tap water, we conducted comparative experiments on TCH solutions, with different compounds added under the same conditions (Figure S5a,b). The results show that most ions had no significant effect on catalytic performance. Even in lake water, it had an excellent degradation performance (Figure S5c). The ZZF2 was soaked in tap water for 2 h and then degraded TCH in DI water, and the performance was not enhanced (Figure S5c). Tap water did not affect the catalyst. The weak pH change may be the reason for the different degradation performance. Figure S5d shows that the effect of pH on the degradation of the ZZF2 is very significant. In an alkaline environment, TCH exists in monoanionic form, while in a neutral environment, TCH exists in neutral form; pH = 7.7 is the critical point [45]. Tap water has a pH of 7.8; therefore, the above results indicate that a small pH difference leads to a large increase in degradation.

As shown in Figure 4d, ZnO is diamagnetism, while ZFO and ZZF2 exhibit weak ferromagnetism. The coercivity of ZFO and ZZF2 is 108 Oe, and the saturation magnetization of ZFO and ZZF2 is 0.53 emu/g and 0.27 emu/g, respectively. For powdered photocatalysts, it is also important to cost-effectively separate them from water [41]. The ZZF2 samples are attracted to magnets in DI water, which means that the ZZF2 samples dispersed in water can be easily recovered using electromagnets. The secondary pollution of water bodies is avoided, and their reusability and economic benefits are improved.

### 3.3. Effect of Temperature on Catalytic Effect

**Figure 5:**
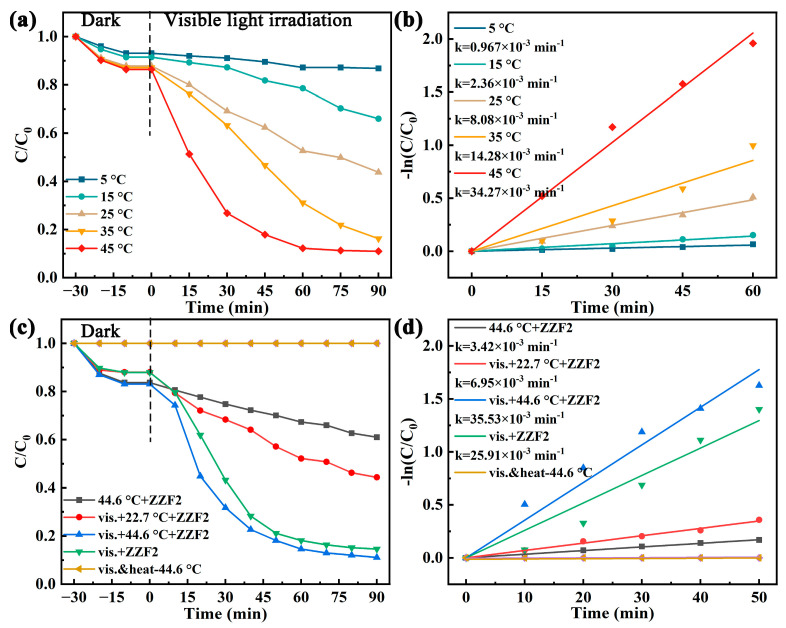
(**a**) Photodegradation performance of TCH degradation and (**b**) corresponding degradation rate constants of ZZF2 at different temperatures. (**c**) Photodegradation performance of TCH degradation and (**d**) corresponding degradation rate constants of ZZF2 at different conditions.

The temperature of the system gradually increases as the catalytic reaction progresses. To determine the effect of temperature on the catalytic performance of ZZF2, the catalytic activity of the reaction system was studied by controlling the temperature at 5 °C, 15 °C, 25 °C, 35 °C, and 45 °C. The results show that the degradation effect of ZZF2 on TCH was gradually improved with the increase in temperature (Figure 5a,b).

To explore the role of temperature, visible light, and catalysts in the reaction, we measured the TCH degradation rate under different conditions, and the results are shown in Figure 5c. In the absence of a catalyst, both visible light irradiation and heating cannot degrade TCH. Since the temperature of the reaction system under visible light irradiation can reach 44.6 °C, according to the principle of control variables, the temperature of photothermal catalysis and thermal catalysis is set at 44.6 °C. The degradation rate of TCH via ZZF2 under thermo-assisted photocatalysis was significantly higher than that under room-temperature photocatalysis and thermocatalysis. It can be seen from Figure 5d that the k_TCH_ value of photothermal catalysis is 35.53 × 10^−3^ min^−1^, while those of thermal catalysis and room-temperature photocatalysis are 3.42 × 10^−3^ min^−1^ and 6.59 × 10^−3^ min^−1^, respectively. The catalytic effect that the whole is greater than the sum of its parts is achieved [56]. Thermal energy not only degrades TCH but also promotes the photocatalytic reaction. If the temperature was not controlled, the k_TCH_ values gradually changed from 6.95 × 10^−3^ min^−1^ at 22.7 °C to 35.53 × 10^−3^ min^−1^ at 44.6 °C as the temperature rose during the reaction. The catalytic efficiency increased by 5.1 times.

The photothermal conversion efficiency (ŋ) and heating performance of the ZZF2 sample were further studied. This calculation method is detailed in Text S4. As shown in Figure S6, when the whole system reaches thermal equilibrium, the system temperature of adding the ZZF2 sample can reach 44.6 °C (ŋ = 91.8%); pure DI water can only reach 33.1 °C (ŋ = 27.9%). After adding the ZZF2 sample, the photothermal conversion efficiency was increased by 3.24 times. This shows that the ZZF2 sample can effectively convert the energy in simulated sunlight into thermal and chemical energy.

### 3.4. Analysis of the Photocatalytic Mechanism

**Figure 6:**
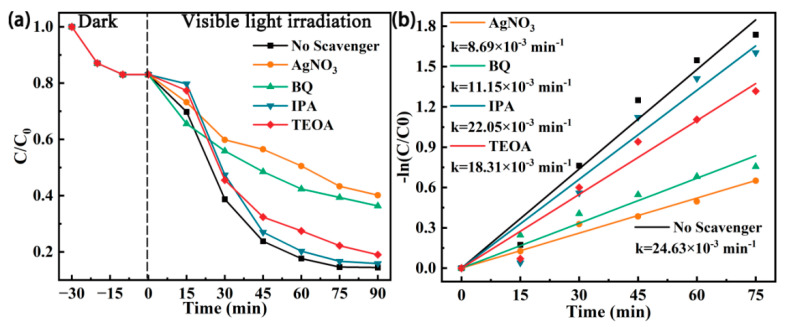
(**a**) The free radical trapping experiments for degradation of TCH over the ZZF2 sample and (**b**) corresponding pseudo-first-order kinetics.

To further understand the photocatalytic mechanism, we conducted free radical trapping experiments on the ZZF2 sample to determine the main active species in the photocatalytic degradation of TCH. As shown in Figure 6a, the isopropanol (IPA), P-benzoquinone (BQ), triethanolamine (TEOA), and silver nitrate (AgNO_3_) were selected as the scavengers for •OH, •O_2_^−^, h^+^, and e^−^, respectively. Their corresponding amount is summarized in Table S3. After adding AgNO_3_ and BQ, the degradation efficiency of TCH was greatly inhibited, indicating that e^−^ and •O_2_^−^ were the main active substances in the photocatalytic degradation of TCH in the ZZF2 sample. The addition of TEOA also affected the degradation rate, indicating that h^+^ also played a role in the degradation process of TCH. However, the addition of IPA had little effect on the TCH degradation rate. To investigate whether •OH is produced during the reaction, the evolution of •OH fluorescence intensity with time was investigated via the TA-PL analysis method (Figure S7a). As time goes on, the fluorescence intensity of •OH gradually increases. This indicates that •OH is produced during the reaction. Subsequently, to determine the effect of reaction conditions on the concentration of free radicals, we studied the fluorescence intensity of hydroxyl radicals under different conditions (Figure S7b). The results show that •OH is hardly produced by thermal catalysis. Under visible light irradiation, the concentration of •OH gradually increases with the increase in temperature. This shows that the promotion effect of temperature on photocatalysis is reflected in the production of more surface-active substances on the catalyst surface. This can be attributed to heat-facilitated carrier separation and migration [13].

**Figure 7:**
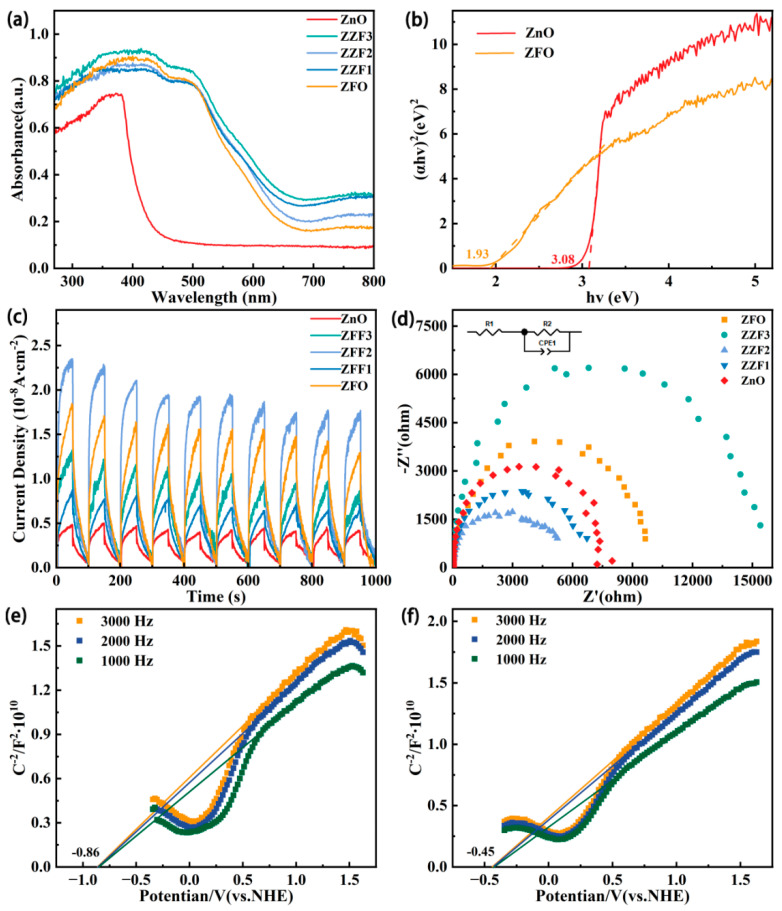
(**a**) The DRS spectra of ZnO, ZFF samples, and ZnFe_2_O_4_. (**b**) Tauc plots of ZnO and ZnFe_2_O_4_. (**c**) Transient photocurrent density responses and (**d**) EIS Nyquist plots irradiated conditions of ZnO, ZFF samples, and ZnFe_2_O_4_. M-S plots of (**e**) ZnFe_2_O_4_ and (**f**) ZnO.

For the thermo-assisted photocatalysis degradation of TCH, the basic steps of catalysis are the same as those of traditional photocatalysis [13,44,57]. The band structure and charge separation capability of catalysts were characterized by DRS, photoluminescence (PL) spectroscopy, and photoelectrochemical measurements. Figure 7a shows the DRS of a series of samples. Compared with pure ZnO, the absorption band edge of the catalyst supported with ZnFe_2_O_4_ has a significant redshift. This means that the composite material has a greater ability to capture visible light. The absorption band edges of ZFO, ZZF1, ZZF2, and ZZF3 experience no significant change. The Tauc plots (Figure 7b) were obtained by converting DRS data through the Kubelka–Munk formula:

Ahυ^1/n^ = A(hυ − E_g_) (2)

For direct bandgap semiconductors, n = 1/2. The band gap energy (E_g_) values of ZnO and ZFO are 3.08 eV and 1.93 eV, respectively, which is consistent with previous reports [44]. PL spectra (Figure S8) show that ZnO has a strong excitation light, but for ZZF2, the excitation light is very small. It is further proven that the heterojunction construction can effectively enhance photogenerated carrier migration.

Figure 7c shows the transient photocurrent density responses of a series of samples. The results show that the transient photocurrent of the ZFF2 sample is significantly higher than those of the ZnO and ZFO samples. Heterojunction formation enhances photogenerated carrier dynamics. The transient photocurrent of ZFO is higher than in the ZZF1 and ZZF3 samples, but its catalytic efficiency is lower than in the ZZF1 and ZZF3 samples. This shows that the band structure of pure ZFO samples is not suitable, and the photogenerated carrier’s redox ability is weak. Figure 7d and Figure S9 show electrochemical impedance spectra under visible light irradiation and darkness, respectively. The ZZF2 sample has the smallest arc radius of all the samples. The arc radius of the series of samples is lower under visible light irradiation than in the dark. A smaller arc radius means a smaller charge transfer resistance [54]. Figure 7e,f are Mott–Schottky plots of ZFO and ZnO, respectively. The slope is positive, which means they are all n-type semiconductors. Additionally, the flat-band potential of ZFO and ZnO are located at −0.86 V vs. NHE and −0.45 V vs. NHE, respectively. For n-type semiconductors, their conduction band position (E_CB_) is usually 0.1 V~0.3 V lower than the Fermi level position (E_F_) [58]. Therefore, the position of E_CB_ is situated at −1.06 V vs. NHE for ZFO and −0.65 V vs. NHE for ZnO. Accordingly, the valence band (E_VB_) positions of ZFO and ZnO can be calculated as 0.87 V vs. NHE and 2.43 V vs. NHE, respectively, using the following formula:

E_VB_ = E_CB_ + E_g_ (3)

The ZnFe_2_O_4_/ZnO junction has staggered band configurations. ZFO has higher E_CB_, E_VB_, and E_F_ positions than ZnO. When ZFO and ZnO are in contact, the free electrons of ZFO can transfer to ZnO until their E_F_ is equilibrated. The transfer of free electrons makes the ZFO side positively charged and the ZnO side negatively charged. A built-in electric field is formed on the contact surface of ZnO and ZFO, which causes the band to bend. The presence of the built-in electric field is conducive to the separation of photogenerated electrons of ZnO with ZFO photogenerated holes. Photogenerated carriers with a greater redox capacity are retained to participate in further reactions [59,60].

**Figure 8:**
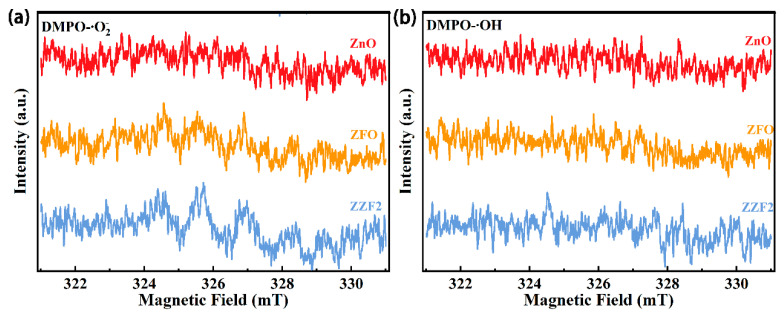
ESR spectra of (**a**) DMPO-•O_2_^−^ and (**b**) DMPO-•OH of ZnO, ZFO, and ZZF2 under visible light irradiation.

To further verify that the photogenerated carrier migration belongs to the Z-scheme charge transfer mode. We identified the active species formed during the photocatalytic reaction using the ESR technique. Under dark conditions, ZnO, ZFO, and ZZF2 cannot produce •OH and •O_2_^−^ (Figure S10). After visible light irradiation (λ ≥ 400 nm) for 30 s, the ZZF2 samples produced •OH and •O_2_^−^ signals (Figure 8a,b). This is consistent with the results of the free radical trapping experiment and TA-PL. The signals of •OH and •O_2_^−^ were not detected by pure ZnO after 30 s of visible light irradiation (Figure 8a,b). This is due to the wide band gap of ZnO, which essentially cannot absorb visible light. After ZFO irradiation, an •O_2_^−^ signal, but no •OH signal, was produced. If the photogenic holes are transferred to ZFO, ZZF2 cannot produce •OH after irradiation. This indicates that the photogenerated holes on ZnO are preserved. This is consistent with the charge transfer path of the Z-scheme heterojunction. At the same time, the •O_2_^−^ signal of the ZZF2 sample is stronger than that of ZFO, indicating that the formation of a heterojunction effectively promotes the separation of photogenerated carriers.

**Figure 9:**
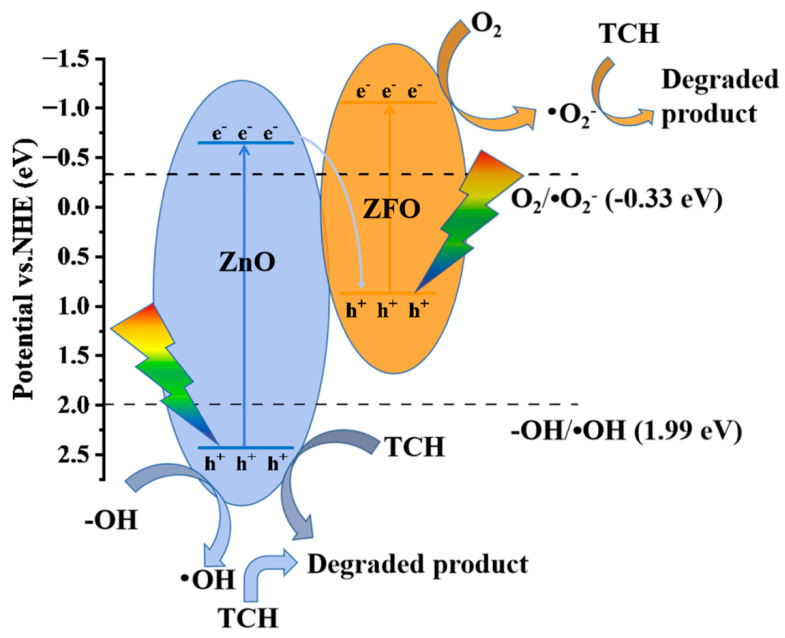
Mechanism of photothermal catalytic degradation of TCH over the ZZF2 heterojunction.

According to the above analysis, the catalytic mechanism of the ZFF2 heterojunction is shown in Figure 9. The catalyst absorbs photons to form electron–hole pairs (Equation (4)). Photogenerated electrons on ZnO recombine with photogenerated holes on ZFO under the action of a built-in electric field. Photogenerated electrons on ZFO and photogenerated holes on ZnO with greater redox capacity are retained and participate in subsequent reactions (Equations (5) and (6)). The nonradiative recombination of photogenerated carriers produces heat. The introduction of thermal energy improves the separation efficiency of photogenerated carriers. Therefore, a large number of hydroxyl radicals and superoxide radicals are produced. Finally, h^+^, •O_2_^−^, and •OH oxidize the TCH (Equation (7)).

ZZF2 + hυ → h^+^ + e^−^ (4)

h^+^ + −OH → •OH (5)

e^−^ + O_2_ → •O_2_^−^ (6)

h^+^, •OH, •O_2_^−^ + TCH → Degraded product (7)

## 4. Conclusions

In summary, the spindle-shaped heterojunction of Z-scheme ZnO/ZnFe_2_O_4_ is synthesized in situ using a solid phase method using MIL-88A(Fe)@Zn as the precursor. The ZZF2 sample has the highest catalytic degradation rate, with 86.3% degradation after 75 min of visible light irradiation. This is attributed to the cooperative action of thermo-assisted photocatalysis and thermocatalysis. Furthermore, the ZZF2 sample also has excellent catalytic activity and reusability in tap water. The Z-scheme charge transfer mechanism promotes carrier transfer dynamics. Superoxide radicals and hydroxyl radicals are the main active species. Moreover, the catalyst can be magnetically recovered from the water. This study provides a reference for the practical application of catalysts to degrade antibiotics in wastewater.

## Data Availability

Raw data are available upon request.

## Supplementary Materials

The following supporting information can be downloaded at: https://www.mdpi.com/article/10.3390/ma16206639/s1, Text S1: Materials; Text S2: Characterizations; Text S3: Photo-electrochemical measurements; Text S4: Photothermal conversion performance; Text S5: Hydroxyl radical concentration test; Table S1: Comparison of the catalytic performance; Table S2: The main elements contained in tap water; Table S3: Scavengers used and oxidizing species quenched; Figure S1: (a) EDS spectra and (b) EDX mapping of ZZF2. (c) Thermogravimetric curve of MIL-88A(Fe)@Zn; Figure S2: (a) The full XPS survey spectra and (b) XPS spectra related to C 1s of ZnFe_2_O_4_, ZnFe_2_O_4_/ZnO heterostructures (sample ZZF2), and ZnO; Figure S3: N_2_ adsorption–desorption isotherms and pore size distribution curves of ZZF2; Figure S4: XRD patterns of the fresh ZZF2 and the used one after the 5th-run reaction; Figure S5: (a) Photodegradation performance and (b) degradation rate constants of the ZZF2 at different compound. (c) Degradation effect of the ZZF2 in different water quality. (d) Photodegradation performance and (e) degradation rate constants of the ZZF2 at different pH; Figure S6: The fitting of parameter B; Figure S7: (a) PL spectra depended on the concentration of •OH radical of ZZF2 under visible light irradiation. (b) •OH radical concentration-related PL spectra of ZZF2 under different conditions with visible light irradiation for an hour; Figure S8: PL spectra of ZnFe_2_O_4_, ZZF2 and ZnO; Figure S9: EIS Nyquist plots under darkness of ZnO, ZZF samples and ZnFe_2_O_4_; Figure S10: ESR spectra of DMPO-•O_2_- and DMPO-•OH of ZnO, ZnFe_2_O_4_ and ZZF2 in the dark. References [61,62,63,64,65,66,67] are cited in the supplementary materials.
