# Supplementary material for: MOF-Derived Spindle-Shaped Z-Scheme ZnO/ZnFe2O4 Heterojunction: A Magnetic Recovery Catalyst for Efficient Photothermal Degradation of Tetracycline Hydrochloride
Source: Materials (Basel). 2023 Oct 11;16(20):6639. doi: 10.3390/ma16206639 (PMC10608245; doi:10.3390/ma16206639)
Supplement: Supplementary file 1 [file materials-16-06639-s001.zip › Supplementary Materials.pdf]

Supplementary Materials

MOF-derived Spindle-shaped Z-scheme $ZnO/ZnFe_2O_4$ Heterojunction: A Magnetic Recovery Catalyst for Efficient Photothermal Degradation of Tetracycline Hydrochloride

Shilong Suo [1], Wenmei Ma [1], Siyi Zhang [1], Ziwu Han [1], Yumin Wang [1], Yuanyuan Li [1], Yi Xiong [2], Yong Liu [1], Chunqing He [1] and Pengfei Fang [1,*]

[1] Key Laboratory of Nuclear Solid State Physics Hubei Province, School of Physics and Technology, Wuhan University, Wuhan 430072, China; 2022202020075@whu.edu.cn (S.S.)
[2] Department of Microelectronics, School of Mathematical & Physical Sciences, Wuhan Textile University, Wuhan 430073, China
* Correspondence: fangpf@whu.edu.cn

Text S1. Materials

The reagents and chemicals were analytical grades with no further purification before use. All of the following reagents were offered by Sinopharm Chemical Reagent Co., Ltd: ferric chloride hexahydrate ($FeCl_3·6H_2O$), fumaric acid ($C_4H_4O_4$), dimethyl formamide (DMF), zinc nitrate hexahydrate ($Zn(NO_3)_2·6H_2O$), isopropyl alcohol (IPA), terephthalic acid (TA), triethanolamine (TEOA).

Tetracycline hydrochloride (TCH) was purchased from Aladdin Industrial Corporation. P-benzoquinone (BQ) was purchased from Shanghai Macklin Biochemical Co., Ltd.

The deionized water (DI water, 18.3 MΩ·cm resistivity) was produced from the deionizer (PGDX-S-10B, Pinguanwater Co, Ltd., China). The tap water is provided by Wuhan Water Bureau. Lake water is taken from East Lake in Wuhan, China

Text S2. Characterizations

X-ray diffraction (XRD) measurements were collected on a Rigaku-Smart Lab equipped with D/tex Ultra250 detector with 3kW X-ray generator, and recorded from 10° to 80° with the scanning rate of 6°/min. X-ray photoelectron spectra (XPS) were recorded on a Thermo 163 Fisher ESCALAB 250Xi system. Scanning electron microscopy (SEM) images and EDX data were recorded on a FEI Quanta 250 FEG instrument. High-resolution transmission electron microscopy (HRTEM) images were recorded on JEM-2100 electron microscope (JEOL JEM-2100Plus). The reflectance spectra of all samples over the 250-800 nm range were gained by a UV-Visible spectrometer equipped with a Labsphere diffuse reflectance accessory (UV-2550, Shimadzu), using $BaSO_4$ as the reference standard. The hysteresis loop was measured by a comprehensive physical property test system (PPMS-9, Quantum Design). The steady-state photoluminescence (PL) spectra of the sample at room temperature were studied with a fluorescence spectrophotometer (Hitachi 4600). The BET surface area (SBET) and the corresponding pore size distribution of the sample was investigated using N_2 adsorption-desorption isotherms (77 K) with apparatus (JW-BK 122W). Before starting the experiment, the test tubes (with lids) and samples are dried in the flowing-gas degassing unit.

Text S3. Photo-electrochemical measurements

The photoelectrochemical measurements (PEC) performances of as-prepared photocatalyst were examined in a 3-arm PEC cell on electrochemical workstation (CSStudio5, CorrTest) with the photocatalyst as working electrode, a Pt slice as the counter electrode, and a saturated calomel electrode (SCE) as the reference.

$$E_{NHE} = E_{SCE} + 0.05916 \times pH + 0.241\ (pH = 7) \qquad (S1)$$

The working electrode was prepared via spin-coated slurry onto 1.0 cm × 2.0 cm Indium tin oxide (ITO) glass followed by drying naturally. The slurry was designed by ultrasonically dissolving 10 mg of the photocatalyst and 80 μL (5%) Nafion solution into 1 mL isopropanol. A 300 W Xe lamp with a 400 nm cut-off filter was used as a light source. When 0.5 M Na_2SO_4 aqueous solution as electrolyte, the transient photocurrent-time (I-t) tests with an electrical bias potential of 0.1 eV vs. SCE, Mott-Schottky (M-S) measurements at the frequency of 1000, 2000 and 3000 Hz with the scanning rate of 0.02 V/s and voltage ranging from -1 ~ 1 V and the linear sweep voltammetry (LSV) were performed with a scanning rate of 10 mV/s and the hydrogen evolution voltage ranging from -1 ~ 0 V. In the 0.1 M Na_2S and 0.1 M Na_2SO_3 mixed solution, the electrochemical impedance spectroscopy (EIS) measurements were performed under an ac amplitude of 10 mV and the frequency range from 10^{-1} Hz to 10^5 Hz.

Text S4. Photothermal conversion performance

The photothermal conversion performance can be described by the photothermal conversion efficiency (ŋ), which can be calculated using the following equation [S1]:

$$ŋ = \frac{(Teq - Tam)mcB}{AS} \qquad (S2)$$

where c, m, A, S, Tam, and Teq denote the specific heat capacity, mass of the base liquid, total area of heat dissipation, light intensity, ambient temperature, and equilibrium temperature, respectively. For **Eq. S2** in the manuscript, c, m, A, and S were 4.18×10^3 $J\cdot g^{-1}\cdot °C^{-1}$, 48.6 g, 86.39 cm^2, and 564.33 $W\cdot m^{-2}$, respectively. The light intensity was measured by the five-hole method. Parameter B is calculated from **Eqs. 3-5**, and the results are shown in **Figure S6**. Here, t1 is the parameter of the fitting equation with A1*exp (-x/t1) +y0.

$$B \equiv \frac{hA_{dis}}{\sum_i m_i c_i} \qquad (S3)$$

$$\frac{T(t) - T_{am}}{T_{eq} - T_{am}} = \exp(-Bt) \qquad (S4)$$

$$B = 1/t1 \qquad (S5)$$

Text S5. Hydroxyl radical concentration test

The 20 mg catalyst was dispersed in 50 ml aqueous solution containing 2×10^{-3} M NaOH and 5×10^{-4} M TA. After reacting for a period of time according to the required conditions, take 3 ml supernatant. The hydroxyl content can be determined by measuring the luminescence intensity at 425 nm.

Table S1. Comparison of the catalytic performance.

Catalyst	Concentration （mg/L）	Catalyst dosage (g/L)	Light source	Time (min)	Removal rate (%)	Degradation Rate * (%/min)	Ref
ZFF2	100.0	0.400	300 W Xe lamp (λ≥400 nm)	75.0	86.3	0.288	This work
ZO_v/ZFO_v 500	88.8	0.400	300 W Xe lamp (λ≥420 nm)	120.0	82.0	0.152	[S2]
Fe_2O_3/g-C_3N_4	10.0	0.500	500 W xenon lamp (λ≥420 nm)	120.0	73.8	0.012	[S3]
Bi/ZF/BFT	44.4	0.400	300 W Xe lamp (λ≥420 nm)	60.0	81.5	0.151	[S4]
CdS/NC−T	40.0	0.200	300 W Xe–arc lamp (λ≥420 nm)	60.0	83.0	0.277	[S5]
$Ag_3PO_4/CuBi_2O_4$	20.0	0.500	300 W xenon lamp (λ≥420 nm)	60.0	75.0	0.050	[S6]
Ag@g-C_3N_4@$BiVO_4$	20.0	0.300	300 W xenon lamp (λ≥420 nm)	60.0	82.8	0.092	[S7]
ZnO-$ZnFe_2O_4$-60	25.0	0.150	500 W Halogen lamp	240.0	93.68	0.065	[S8]
0.6-ZBO	40.0	0.200	300 W Xe lamp (λ≥400 nm)	90.0	85.6	0.186	[S9]

*Degradation Rate=Concentration×Removal rate/(Catalyst dosage×Time)

Table S2. The main elements contained in tap water

Detection index	Detection result	Detection index	Detection result	Detection index	Detection result
As (mg/L)	0.0014	Cd (mg/L)	<0.0001	Cr^{6+} (mg/L)	<0.004
Ba (mg/L)	0.056	B (mg/L)	0.065	Mo (mg/L)	0.0015
Zn (mg/L)	<0.001	Al (mg/L)	0.088	pH	7.82
Mn (mg/L)	0.0001	Fe (mg/L)	0.011	Cu (mg/L)	0.0008
Chlorate (mg/L)	0.08	Chloride (mg/L)	32.8	Sulfate (mg/L)	52.2
Permanganate (mg/L)	0.92	Ammonia (mg/L)	<0.02	Free chlorine (mg/L)	0.65
Fluoride (mg/L)	0.16	Nitrate (mg/L)	2.15	Na (mg/L)	28.6
Ni (mg/L)	0.0004	Ag (mg/L)	<0.0001		

Table S3. Scavengers used and oxidizing species quenched.

Scavengers	ROSs quenched	Scavenger dosage
Isopropanol (IPA)	•O^{2-}	0.1M
Benzoquinone(BQ)	•OH	0.1 mM
Triethanolamine(TEOA)	h^+	0.1M
Silver nitrate ($AgNO_3$)	e^-	0.1M

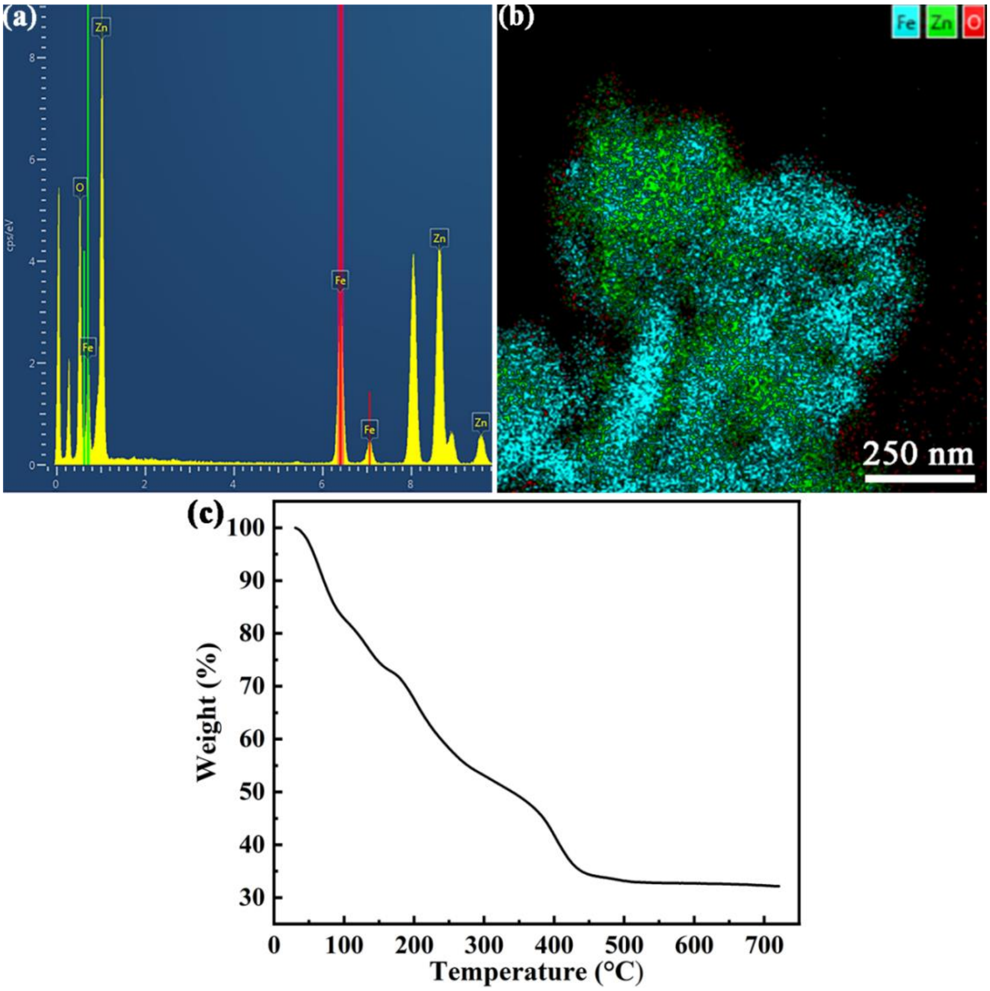

Figure S1. (a) EDS spectra and (b) EDX mapping of ZZF2. (c) Thermogravimetric curve of MIL-88A(Fe)@Zn.

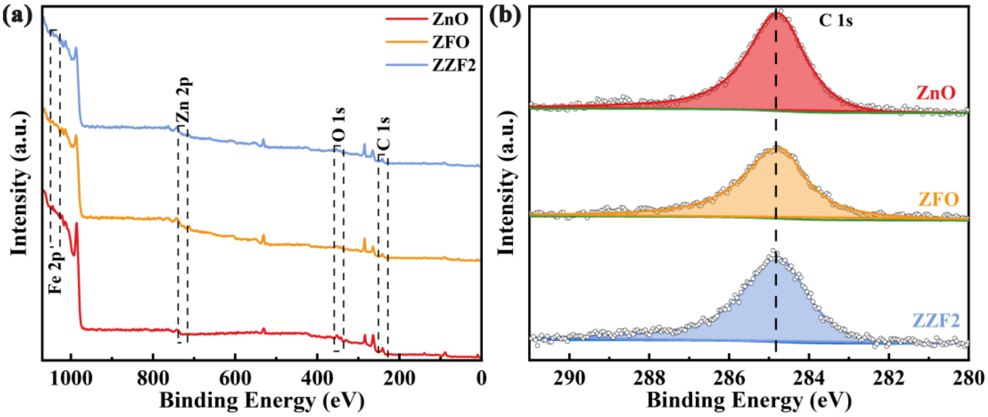

Figure S2. (a) The full XPS survey spectra and (b) XPS spectra related to C 1s of $ZnFe_2O_4$, $ZnFe_2O_4$/ZnO heterostructures (sample ZZF2), and ZnO.

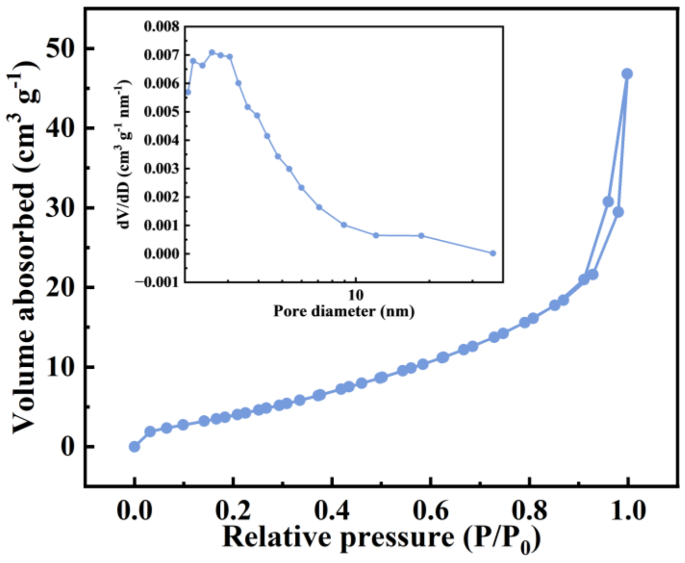

Figure S3. N_2 adsorption–desorption isotherms and pore size distribution curves of ZZF2.

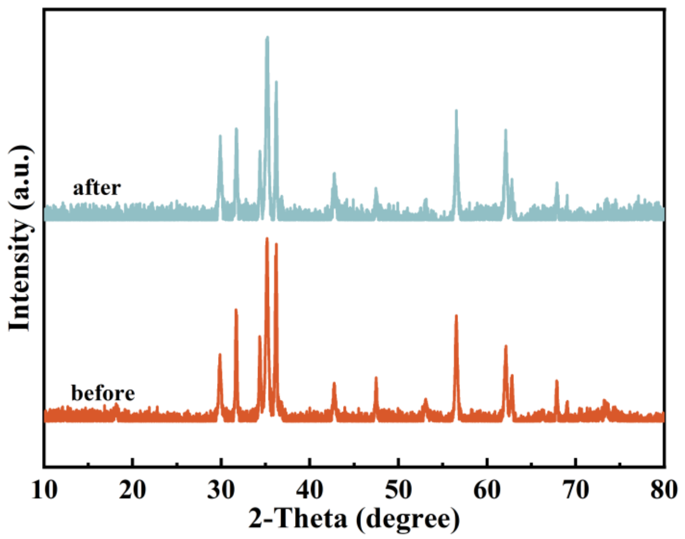

Figure S4. XRD patterns of the fresh ZZF2 and the used one after the 5th-run reaction.

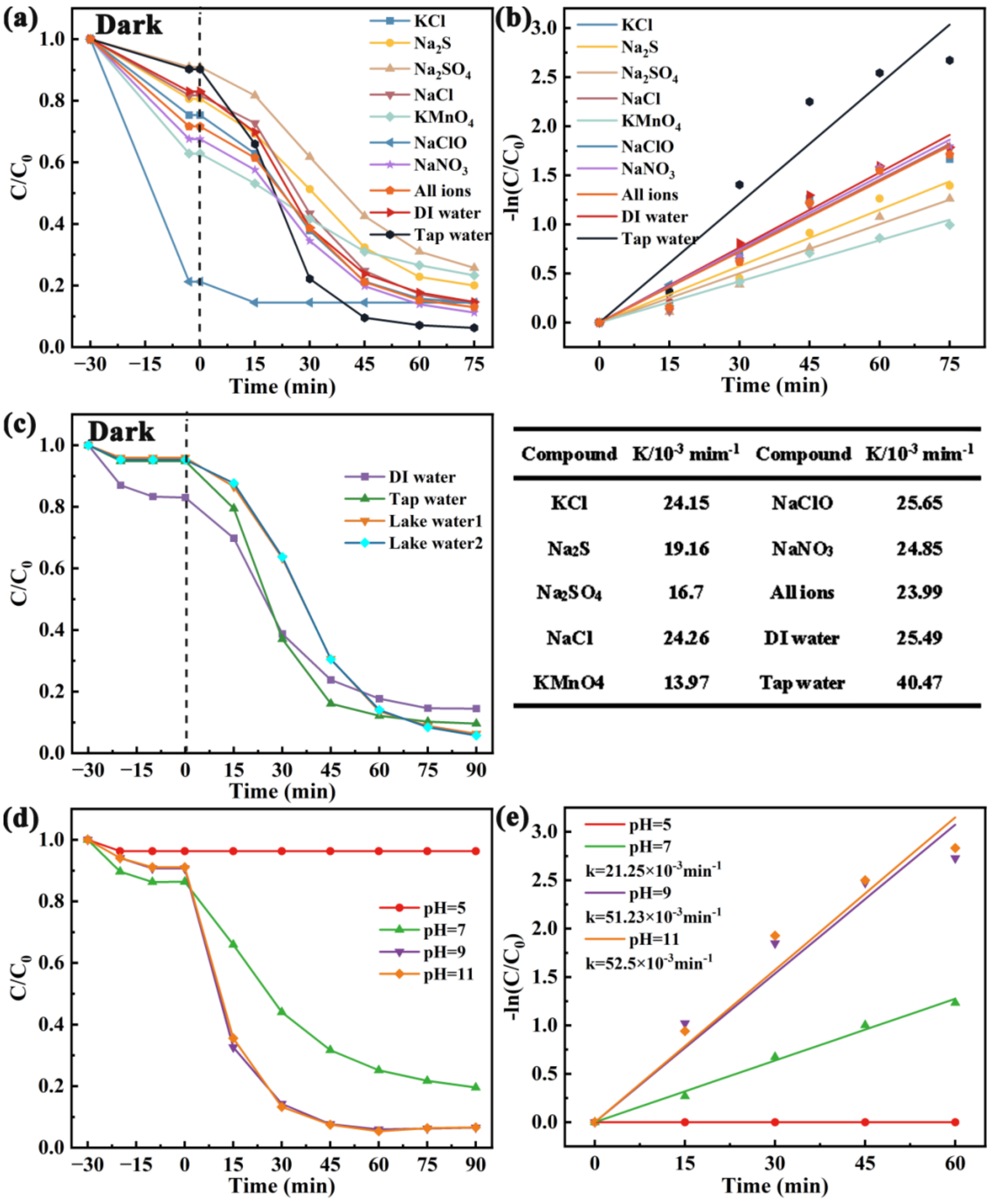

Compound	$K/10^{-3}$ mim^{-1}	Compound	$K/10^{-3}$ mim^{-1}
KCl	24.15	NaClO	25.65
Na_2S	19.16	$NaNO_3$	24.85
Na_2SO_4	16.7	All ions	23.99
NaCl	24.26	DI water	25.49
KMnO4	13.97	Tap water	40.47

Figure S5. (a) Photodegradation performance and (b) degradation rate constants of the ZZF2 at different compound. (c) Degradation effect of the ZZF2 in different water quality. (d) Photodegradation performance and (e) degradation rate constants of the ZZF2 at different pH.

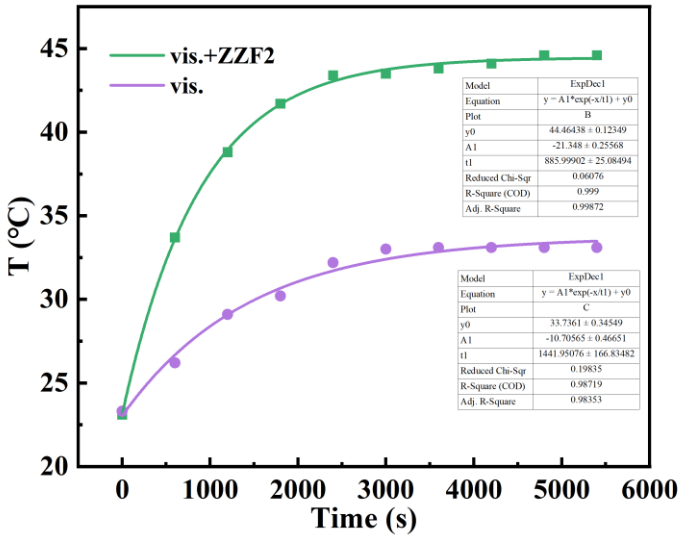

Figure S6. The fitting of parameter B.

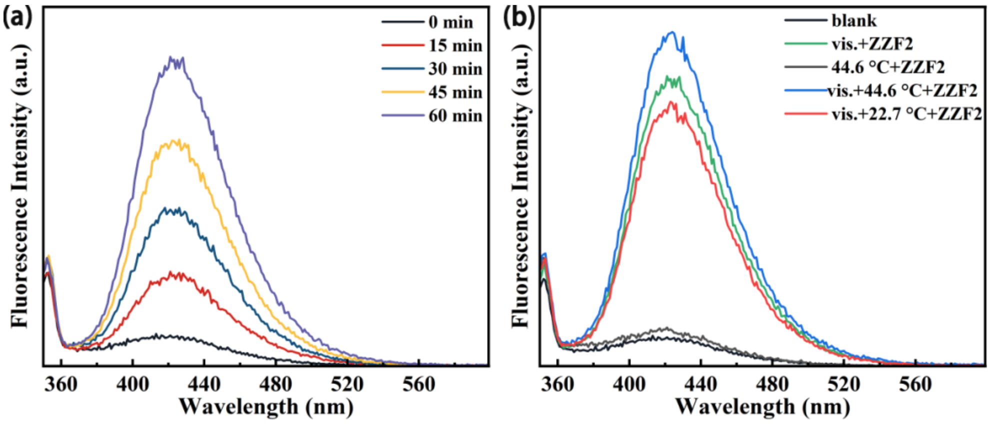

Figure S7. (a) PL spectra depended on the concentration of •OH radical of ZZF2 under visible light irradiation. (b) •OH radical concentration-related PL spectra of ZZF2 under different conditions with visible light irradiation for an hour.

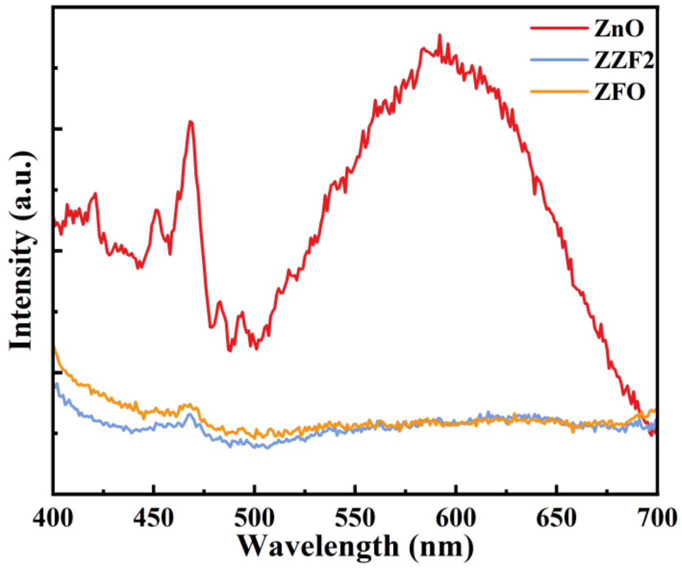

Figure S8. PL spectra of $ZnFe_2O_4$, ZZF2 and ZnO.

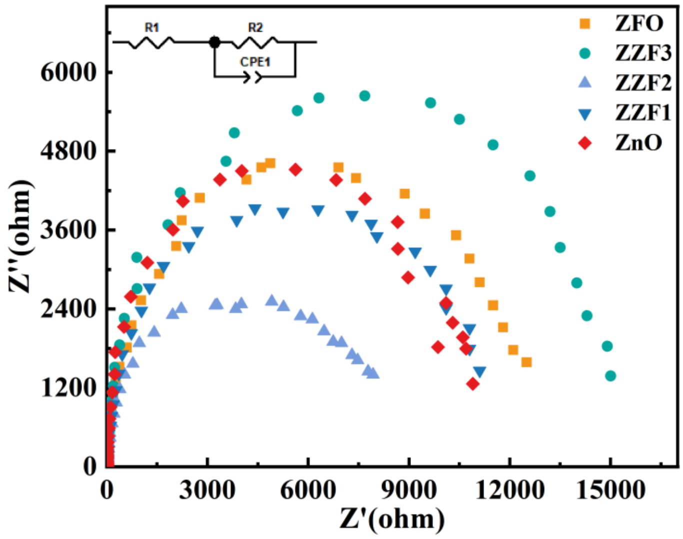

Figure S9. EIS Nyquist plots under darkness of ZnO, ZZF samples and $ZnFe_2O_4$.

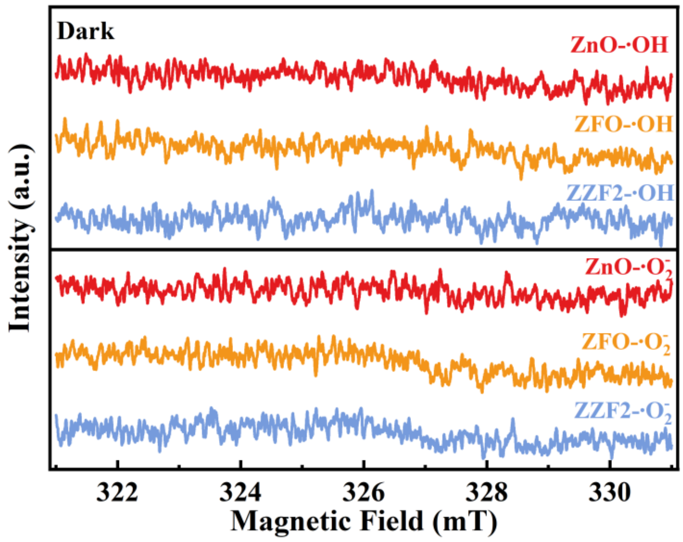

Figure S10. ESR spectra of DMPO -•O2- and DMPO -•OH of ZnO, $ZnFe_2O_4$ and ZZF2 in the dark.

References

S1. D.H. Zhu, L. Cai, Z.Y. Sun, A. Zhang, P. Heroux, H. Kim, W. Yu, Y.A. Liu, Efficient degradation of tetracycline by RGO@black titanium dioxide nanofluid via enhanced catalysis and photothermal conversion, *Sci. Total Environ.*, **2021,** *787*. https://doi.org/10.1016/j.scitotenv.2021.147536.

S2. K. Zhang, H.Y. Cao, A. Dar, D.Q. Li, L.A. Zhou, C.Y. Wang, Construction of oxygen defective ZnO/$ZnFe_2O_4$ yolk-shell composite with photothermal effect for tetracycline degradation: Performance and mechanism insight, *Chin. Chem. Lett.*, **2023,** *34(1)*. https://doi.org/10.1016/j.cclet.2022.03.031.

S3. C. Li, S. Yu, H. Che, X. Zhang, J. Han, Y. Mao, Y. Wang, C. Liu, H. Dong, Fabrication of Z-Scheme Heterojunction by Anchoring Mesoporous γ-Fe_2O_3 Nanospheres on g-C_3N_4 for Degrading Tetracycline Hydrochloride in Water, *ACS Sustain. Chem. Eng.*, **2018,** *6(12),* 16437-16447. https://doi.org/10.1021/acssuschemeng.8b03500.

S4. K. Zhang, D. Li, Q. Tian, H. Cao, F. Orudzhev, I.A. Zvereva, J. Xu, C. Wang, Recyclable 0D/2D $ZnFe_2O_4$/$Bi_5FeTi_3O_{15}$ S-scheme heterojunction with bismuth decoration for enhanced visible-light-driven tetracycline photodegradation, *Ceram. Int.*, **2021,** *47(12),* 17109-17119. https://doi.org/https://doi.org/10.1016/j.ceramint.2021.03.020.

S5. H.L. Cao, F.Y. Cai, K. Yu, Y.Q. Zhang, J. Lu, R. Cao, Photocatalytic Degradation of Tetracycline Antibiotics over CdS/Nitrogen-Doped-Carbon Composites Derived from in Situ Carbonization of Metal-Organic Frameworks, *ACS Sustain. Chem. Eng.*, **2019,** *7(12),* 10847-+. https://doi.org/10.1021/acssuschemeng.9b01685.

S6. W.L. Shi, F. Guo, S.L. Yuan, In situ synthesis of Z-scheme Ag_3PO_4/$CuBi_2O_4$ photocatalysts and enhanced photocatalytic performance for the degradation of tetracycline under visible light irradiation, *Appl. Catal. B-Environ.*, **2017,** *209* 720-728. https://doi.org/10.1016/j.apcatb.2017.03.048.

S7. F. Chen, Q. Yang, Y.L. Wang, J.W. Zhao, D.B. Wang, X.M. Li, Z. Guo, H. Wang, Y.C. Deng, C.G. Niu, G.M. Zeng, Novel ternary heterojunction photcocatalyst of Ag nanoparticles and g-C_3N_4 nanosheets co-modified $BiVO_4$ for wider spectrum visible-light photocatalytic degradation of refractory pollutant, *Appl. Catal. B-Environ.*, **2017,** *205* 133-147. https://doi.org/10.1016/j.apcatb.2016.12.017.

S8. C. Akshhayya, M.K. Okla, W.H. Al-Qahtani, M.R. Rajeshwari, A. Mohebaldin, Y.A. Alwasel, W. Soufan, M.A. Abdel-Maksoud, H. AbdElgawad, L.L. Raju, A.M. Thomas, S.S. Khan, Novel $ZnFe_2O_4$ decorated on ZnO nanorod: Synergistic photocatalytic degradation of tetracycline, kinetics, degradation pathway and antifungal activity, *J. Environ. Chem. Eng.*, **2022,** *10(3)*. https://doi.org/10.1016/j.jece.2022.107673.

S9. J.H. Luo, Y.H. Wu, M.Z. Jiang, A.H. Zhang, X.Y. Chen, Y.L. Zeng, Y.H. Wang, Y.L. Zhao, G.J. Wang, Novel $ZnFe_2O_4$/BC/ZnO photocatalyst for high-efficiency degradation of tetracycline under visible light irradiation, *Chemosphere*, **2023,** *311*. https://doi.org/10.1016/j.chemosphere.2022.137041.
